# Severe cutaneous adverse reactions associated with systemic ivermectin: A pharmacovigilance analysis

**DOI:** 10.1111/1346-8138.16398

**Published:** 2022-04-27

**Authors:** David Bomze, Eli Sprecher, Shamir Geller

**Affiliations:** ^1^ Division of Dermatology Tel Aviv Sourasky Medical Center and Sackler Faculty of Medicine Tel Aviv Israel

**Keywords:** FDA adverse event reporting system, ivermectin, severe cutaneous adverse event, Stevens–Johnson syndrome, toxic epidermal necrolysis

## Abstract

Despite poor evidence, the antiparasitic ivermectin has been advocated as a potential COVID‐19 therapy. This has led to a rise in calls to poison‐control centers by people self‐medicating with ivermectin, which is sold over the counter for veterinary uses. We aimed to investigate the association between severe cutaneous adverse reactions (SCARs) and ivermectin. Postmarketing data from the FDA Adverse Event Reporting System (FAERS), gathered between 2014 and 2021, was employed to detect disproportional signals of SCARs following systemic ivermectin therapy. The reporting odds ratio (ROR) was used to quantify the strength of association, while adjusting for age, sex, and region. The search yielded 517 reports of systemic ivermectin (median age 54 years, 46.8% female), of which 25 (4.8%), 81 (15.7%), and 411 (79.5%) were classified as SCARs, nonsevere cutaneous adverse events (AEs), or noncutaneous AEs, respectively. The regional distribution differed between SCAR reports (32.0% from Africa and 12.0% from North America) compared with other AEs, which originated from North America in over half of cases. The most common SCARs were toxic epidermal necrolysis (seven cases), Stevens–Johnson syndrome (seven cases), and drug reaction with eosinophilia and systemic symptoms (four cases). Five SCAR cases (20.0%) resulted in death and 12 (48.0%) lead to hospitalization. There was a strong safety signal for any SCAR (adjusted ROR 3.34, 95% confidence interval [CI] 2.17–5.12) and toxidermias (adjusted ROR 7.08, 95% CI 4.23–11.84). This study suggests that ivermectin is associated with SCARs on rare occasions. Dermatologists should be aware of this given the increase in ivermectin misuse.

## 1 INTRODUCTION

Parallel to the development of novel therapies, there have been multiple attempts to treat COVID‐19 using repurposed drugs, including corticosteroids, remdesivir, and tocilizumab. Some candidates have raised much controversy, especially hydroxychloroquine, which had been initially widely used but was eventually shown to be ineffective in several randomized trials.^1^ The antiparasitic ivermectin recently gained support as a new therapeutic agent for COVID‐19.^2^ However, the World Health Organization, US Food and Drug Administration, European Medicines Agency, and Merck, the drug's manufacturer, all advised against using ivermectin outside clinical trials^3^ since high dosages would be required for antiviral activity, increasing the risk of adverse events (AEs). Although ivermectin has a good safety profile in the recommended dosages, some rare events may go undetected in clinical trials or retrospective cohorts. Therefore, postmarketing surveillance data, aggregated from spontaneous reports, is a useful tool for investigating rare AEs and severe cutaneous adverse reactions (SCARs) in particular.

## 2 METHODS

This was a retrospective analysis of postmarketing safety data from the FDA Adverse Event Reporting System (FAERS), collected between July 2014 and June 2021. FAERS utilizes the Medical Dictionary for Regulatory Activities (MedDRA), a hierarchical and rigorously maintained standardized terminology of medical concepts. Duplicate reports were recognized as those with overlaps in at least three out of four key fields: date, age, gender, and country.^4^ We selected reports in which orally administered ivermectin was the primary suspect and adopted a conservative approach by assuming topical ivermectin was used in cases where the indication was lice infestation, acarodermatitis, or rosacea and the route of administration was unknown. Acarodermatitis is a MedDRA term that may be used to indicate any of the following conditions: acarodermatitis, Norwegian scabies, *Sarcoptes scabiei* infestation, scabies, and scabies infestation.^5^ Severe cutaneous adverse reactions (SCARs) were defined according to the Standardized MedDRA Query (SMQ). If a case reported both Stevens–Johnson syndrome (SJS) and toxic epidermal necrolysis (TEN), it was considered only as TEN, since these conditions lie on a disease spectrum.

We employed a disproportionality analysis to estimate safety signals from spontaneous AE reports,^4^ using the validated reporting odds ratio (ROR), which is the odds of reporting a specific AE versus all other AEs under a certain product, compared to all other products available in FAERS. The ROR is analogous to the odds ratio in a case‐control study where the exposure is ivermectin and the outcome is reporting of SCAR. Multivariate logistic regression was performed using age, sex, region, and ivermectin use as covariates for calculating the adjusted odds of SCAR. The Kruskal–Wallis or *χ* ^2^ tests were used to compare continuous or categorical data across groups, respectively, setting a significance level of 0.05. All statistical analyses were performed in R, version 3.5.0. Because data are publicly available, ethics approval was not required.

## 3 RESULTS

The search yielded 517 reports of systemic ivermectin (median age 54 years, interquartile range (IQR] 33.5–70.5 years, 46.8% female), of which 25 (4.8%), 81 (15.7%), and 411 (79.5%) were classified as SCARs, nonsevere cutaneous AEs, or noncutaneous AEs, respectively (Table 1). Within SCAR cases, the median age was 43 years (IQR 29–69.25 years) and 48.0% were female. The median dosage was 9 mg (IQR 3.75–12 mg) and the median time to onset was 3 days (IQR 1.5–7.25 days), although this information was missing for more than half of cases. Age and sex did not differ between the three groups (*P* = 0.355 for age comparison, *P* = 0.178 for sex comparison), as well as dose and time to onset (*P* = 0.075 and *P* = 0.270, respectively). SCAR cases originated from Africa (8/25 cases, 32.0%), Europe (7/25 cases, 28.0%), Asia (6/25 cases, 24.0%), and North America (3/25 cases, 12.0%). This was in contrast to the other cases, which originated from North America in over 50% of cases, but from Africa in less than 10% of cases (*χ* ^2^ = 25.85, *P* < 0.001).

**TABLE 1:** All cases of systemic ivermectin reported to FAERS, 2014 to 2021

	Severe cutaneous adverse reactions	Nonsevere cutaneous adverse events	Noncutaneous adverse events	*P* ^d^
Total, *n*	25	81	411	
Age, years (median [IQR])	43 [29, 69.25]	50 [31, 66]	55 [35, 72]	0.355
Sex, *n* (%)				
Female	12 (48.0)	41 (50.6)	189 (46.0)	0.178
Male	12 (48.0)	23 (28.4)	156 (38.0)	
Unknown	1 (4.0)	17 (21.0)	66 (16.1)	
Region, *n* (%)				
Africa	8 (32.0)	8 (9.9)	36 (8.8)	0.004
Asia	6 (24.0)	11 (13.6)	57 (13.9)	
Europe	7 (28.0)	14 (17.3)	69 (16.8)	
North America	3 (12.0)	45 (55.6)	238 (57.9)	
Oceania	0 (0.0)	0 (0.0)	4 (1.0)	
South America	0 (0.0)	1 (1.2)	2 (0.5)	
Unknown	1 (4.0)	2 (2.5)	5 (1.2)	
Dose, mg (median [IQR])	9 [3.75, 12]	10.5 [6, 12]	12 [9, 12]	0.075
Onset, days (median, [IQR])	3 [1.5, 7.25]	3.5 [1, 10.5]	2 [0, 7]	0.270
Indication, *n* (%)^a^				
Acarodermatitis	12 (48.0)	32 (39.5)	110 (26.8)	0.490
Helminthic infection	8 (32.0)	13 (16.0)	119 (29.0)	
Infection prophylaxis	1 (4.0)	1 (1.2)	21 (5.1)	
Lice infestation	1 (4.0)	2 (2.5)	6 (1.5)	
COVID‐19	0 (0.0)	1 (1.2)	27 (6.6)	
Unknown	3 (12.0)	24 (29.6)	91 (22.1)	
Adverse events, *n* (%)^b^	Stevens–Johnson syndrome; 7 (28)	Pruritus; 31 (38.3)	Altered mental status; 26 (6.3)	
	Toxic epidermal necrolysis; 7 (28)	Rash; 16 (19.8)	Ocular/visual AE; 23 (5.6)	
	DRESS; 4 (16)	Angioedema; 11 (13.6)	Nausea/vomiting; 21 (5.1)	
	Oral blister/ulcer; 3 (12)	Acarodermatitis; 6 (7.4)	Balance disorder; 20 (4.9)	
	Erythema multiforme; 2 (8)	Erythema; 6 (7.4)	Headache; 20 (4.9)	
	Skin exfoliation; 2 (8)	Alopecia; 4 (4.9)	Encephalopathy; 18 (4.4)	
	AGEP; 1 (4)	Eczema; 4 (4.9)	Coma; 17 (4.1)	
	Bullous dermatitis; 1 (4)	Herpes zoster; 3 (3.7)	Elevated LFT; 17 (4.1)	
	Exfoliative dermatitis; 1 (4)	Pain of skin; 3 (3.7)	Psychiatric disorders; 17 (4.1)	
	Pemphigoid; 1 (4)	Skin disorder; 3 (3.7)	Dizziness; 13 (3.2)	
	SDRIFE; 1 (4)			
	Toxic skin eruption; 1 (4)			
Outcome, *n* (%)^c^				
Death	5 (20.0)	4 (4.9)	36 (8.8)	0.001
Life‐threatening	1 (4.0)	1 (1.2)	5 (1.2)	
Hospitalization	12 (48.0)	20 (24.7)	93 (22.6)	
Disability	3 (12.0)	2 (2.5)	14 (3.4)	
Other serious	4 (16.0)	12 (14.8)	80 (19.5)	
Unknown	0 (0.0)	42 (51.9)	183 (44.5)	
Reporter's occupation, *n* (%)				
Physician	14 (56.0)	24 (29.6)	117 (28.5)	0.004
Consumer	4 (16.0)	38 (46.9)	124 (30.2)	
Pharmacist	2 (8.0)	5 (6.2)	45 (10.9)	
Other health professional	4 (16.0)	14 (17.3)	103 (25.1)	
Unknown	1 (4.0)	0 (0.0)	22 (5.4)	
Year of reporting, *n* (%)				
2014	1 (4.0)	16 (19.8)	20 (4.9)	<0.001
2015	5 (20.0)	14 (17.3)	61 (14.8)	
2016	1 (4.0)	8 (9.9)	36 (8.8)	
2017	2 (8.0)	15 (18.5)	66 (16.1)	
2018	4 (16.0)	5 (6.2)	57 (13.9)	
2019	9 (36.0)	13 (16.0)	69 (16.8)	
2020	2 (8.0)	5 (6.2)	65 (15.8)	
2021	1 (4.0)	5 (6.2)	37 (9.0)	

Abbreviations: AE, adverse event; AGEP, acute generalized exanthematous pustulosis; DRESS, drug reaction with eosinophilia and systemic symptoms; FAERS, FDA adverse event reporting system; IQR, interquartile range; LFT, liver function tests; SCAR, severe cutaneous adverse reaction; SDRIFE, symmetrical drug‐related intertriginous and flexural exanthema.

^a^ Only indications with at least five cases are presented. Therefore, rows may not sum up to 100%.

^b^ Only AEs that comprise at least 3% of cases are presented. Therefore, rows may not sum up to 100%.

^c^ The primary reason of death, life‐threatening scenario, hospitalization, or disability may be other co‐occurring AEs.

^d^ The Kruskal–Wallis or *χ* ^2^ tests were used to compare continuous or categorical data across the three groups, respectively.

**FIGURE 1:**
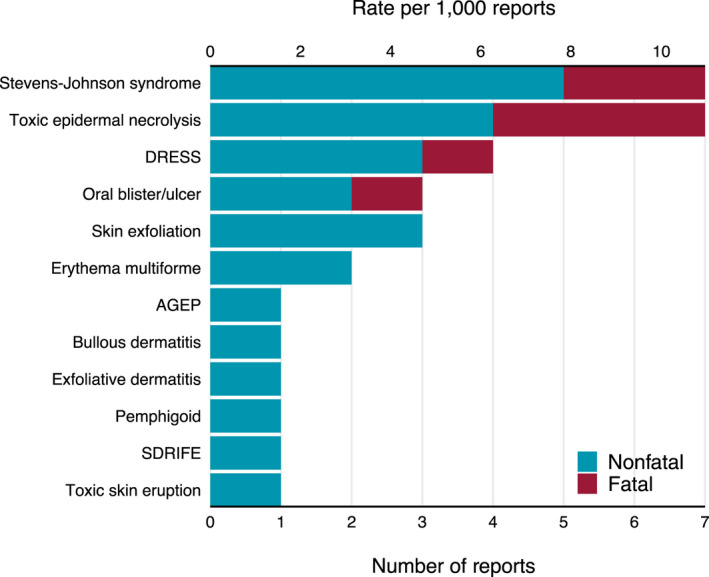
SCARs associated with systemic ivermectin reported to FAERS, 2014 to 2021. AGEP, acute generalized exanthematous pustulosis; DRESS, drug reaction with eosinophilia and systemic symptoms; FAERS, FDA adverse event reporting system; SCAR, severe cutaneous adverse reaction; SDRIFE, symmetrical drug‐related intertriginous and flexural exanthema. *There were overall five SCAR cases where death was reported. There were two cases of Stevens–Johnson syndrome and three cases of toxic epidermal necrolysis, out of which one reported concomitant oral blister/ulcer and another reported concomitant DRESS

The most common indications were acarodermatitis due to mite infestation and helminthic infections, including filariasis, onchocerciasis, and strongyloidiasis. In a few cases concomitant drugs previously associated with SCARs were reported as secondary suspects (two cases of amoxicillin and one case of valproic acid). The most common SCARs were TEN, SJS, and drug reaction with eosinophilia and systemic symptoms (DRESS), occurring in seven (28.0%), seven (8.0%), and four (16.0%) cases, respectively (Figure 1). Among SCAR cases, five (20.0%) resulted in death and 12 (48.0%) in hospitalization. The most common nonsevere cutaneous AEs were pruritus (31 cases, 38.3%), rash (16 cases, 19.8%), and angioedema (11 cases, 13.6%). Death was reported in four cases (4.9%) of nonsevere cutaneous AEs, but was due to other co‐occurring AEs such as sepsis or other organ failure.

Disproportionality analysis (Table 2) revealed that ivermectin was significantly associated with reporting of any SCAR (crude ROR 4.34, 95% confidence interval [CI] 2.84–6.64). The association was still evident after adjusting for age, sex, and region (adjusted ROR 3.34, 95% CI 2.17–5.12, *P* < 0.001). Age under 60 (adjusted ROR 1.28, 95% CI 1.26–1.29, *P* < 0.001) and female sex (adjusted ROR 1.27, 95% CI 1.25–1.29, *P* < 0.001) were also correlated with SCAR reporting to a lesser degree. When using toxidermias (TEN, SJS, DRESS, and acute generalized exanthematous pustulosis) as a binary outcome, the association with ivermectin was more pronounced (crude ROR 15.4, 95% CI 9.3–25.48, *P* < 0.001), even when correcting for other covariates (adjusted ROR 7.08, 95% CI 4.23–11.84, *P* < 0.001).

**TABLE 2:** Multivariate logistic regression analysis of severe cutaneous adverse reactions reported to FAERS, 2014 to 2021^a^

Covariate	*n*/total cases	Proportion (95% CI)	Crude ROR (95% CI)	Adjusted ROR (95% CI)	*P*
A	Any severe cutaneous adverse reactions				
Ivermectin					
Nonuser	83 156/4445256	0.019 (0.019–0.019)	1 (reference)	1 (reference)	
User	23/301	0.076 (0.051–0.112)	4.34 (2.84–6.64)	3.34 (2.17–5.12)	<0.001
Age, years					
≥60	34 255/2060399	0.017 (0.016–0.017)	1 (reference)	1 (reference)	
<60	48 924/2385158	0.021 (0.020–0.021)	1.24 (1.22–1.26)	1.28 (1.26–1.29)	<0.001
Sex					
Male	30 003/1770624	0.017 (0.017–0.017)	1 (reference)	1 (reference)	
Female	53 176/2674933	0.020 (0.020–0.020)	1.18 (1.16–1.19)	1.27 (1.25–1.29)	<0.001
Region^b^					
North America	45 640/3173953	0.014 (0.014–0.015)	1 (reference)	1 (reference)	
Africa	553/17367	0.032 (0.029–0.035)	2.25 (2.07–2.45)	2.20 (2.02–2.40)	<0.001
Asia	13 007/339807	0.038 (0.038–0.039)	2.73 (2.67–2.78)	2.89 (2.84–2.95)	<0.001
Europe	21 419/768491	0.028 (0.028–0.028)	1.97 (1.93–2.00)	2.03 (2.00–2.06)	<0.001
Oceania	753/37626	0.020 (0.019–0.021)	1.40 (1.30–1.51)	1.44 (1.34–1.55)	<0.001
South America	1807/108313	0.017 (0.016–0.017)	1.16 (1.11–1.22)	1.16 (1.11–1.22)	<0.001
**B**	**Toxidermias: one of toxic epidermal necrolysis, Stevens–Johnson syndrome, DRESS, or AGEP**				
Ivermectin					
Nonuser	16 149/4445256	0.004 (0.004–0.004)	1 (reference)	1 (reference)	
User	16/301	0.053 (0.033–0.085)	15.4 (9.3–25.48)	7.08 (4.23–11.84)	<0.001
Age, years					
≥60	6332/2060399	0.003 (0.003–0.003)	1 (reference)	1 (reference)	
<60	9833/2385158	0.004 (0.004–0.004)	1.34 (1.3–1.39)	1.49 (1.44–1.54)	<0.001
Sex					
Male	7238/1770624	0.004 (0.004–0.004)	1 (reference)	1 (reference)	
Female	8927/2674933	0.003 (0.003–0.003)	0.82 (0.79–0.84)	1.0031 (0.9721–1.0351)	0.844
Region^b^					
North America	3749/3173953	0.001 (0.001–0.001)	1 (reference)	1 (reference)	
Africa	348/17367	0.020 (0.018–0.022)	17.29 (15.48–19.32)	16.12 (14.42–18.02)	<0.001
Asia	3966/339807	0.012 (0.011–0.012)	9.99 (9.55–10.44)	10.33 (9.88–10.81)	<0.001
Europe	7518/768491	0.010 (0.010–0.010)	8.35 (8.03–8.69)	8.49 (8.17–8.84)	<0.001
Oceania	317/37626	0.008 (0.008–0.009)	7.18 (6.4–8.06)	7.23 (6.44–8.11)	<0.001
South America	267/108313	0.002 (0.002–0.003)	2.09 (1.85–2.37)	2.08 (1.84–2.36)	<0.001

Abbreviations: AGEP, acute generalized exanthematous pustulosis; CI, confidence interval; DRESS, drug reaction with eosinophilia and systemic symptoms; FAERS, FDA adverse event reporting system; ROR, reporting odds ratio; SCAR, severe cutaneous adverse reactions.

^a^ The entire FAERS database, excluding cases with missing covariates, was used to calculate a reporting odds ratio (ROR). The ROR is therefore analogous to an odds ratio in a case–control design, where the exposure is the drug (e.g. ivermectin) and the outcome is reporting of an AE of interest (e.g. SCAR or toxidermias).

^b^ Two cases originating from Antarctica were excluded, these did not report any SCARs.

## 4 DISCUSSION

Ivermectin has been used for more than three decades for treating filariasis, onchocerciasis, and strongyloidiasis in humans, as well as other dermatologic conditions such as scabies, lice infestation, or demodicosis. While generally safe at recommended dosages,^6^ ivermectin may lead to pruritus, lymphadenitis, arthralgia, and fever, sometimes as part of the Mazzotti reaction. Although the most serious ivermectin safety concern is rare neurotoxicity,^5^ several cases of SCARs following systemic ivermectin have been reported in recent years.^7^, ^8^, ^9^, ^10^ Our search yielded 25 cases of ivermectin‐related SCARs, mostly SJS, TEN, and DRESS, with a 20% mortality. Disproportionality analysis revealed a significant association between systemic ivermectin and reporting of any SCARs, confirming a recent study of VigiBase, another pharmacovigilance database.^11^ Interestingly, SCARs were reported more in African countries compared to other cutaneous AEs. This could be explained by under‐reporting of milder cutaneous AEs in these countries compared with North American or European countries, or by increased incidence of HIV carriers in Africa,^12^ which is a risk factor for some SCARs, in particular SJS and TEN.^13^

This work has several limitations, inherent to any analysis of spontaneous AE reports, such as missing data, under‐reporting, and lack of clinical information. Another important limitation is indication bias, where cases of pruritus reflect the indication (scabies‐related pruritus), rather than the AE. Moreover, the ROR does not represent the incidence of any particular AE, since the total number of individuals treated with ivermectin is unknown. The data cannot be automatically applied to use in COVID‐19 since no SCARs were recorded in this recent setting. Although we focused on reports in which ivermectin was the primary suspect drug, in three out 25 SCAR cases there was concomitant use of secondary suspects known to cause SCARs, which weakens the possible association. Lastly, a causal relationship between drug exposure and the reported AE cannot be fully ascertained.

A major challenge of the current pandemic is the fight against the COVID‐19 “infodemic”, the dissemination of false or inaccurate information. Unfortunately, misleading data obtained from ivermectin studies with poor methodology continue to amass.^14^ This has led to a recent dramatic surge in calls to US poison‐control centers by people who are self‐medicating with ivermectin and, more alarmingly, self‐dosing, as the drug is sold without prescription for veterinary purposes in the USA.^15^ In view of this emerging situation, dermatologists should be aware of ivermectin‐associated SCARs.

## CONFLICT OF INTEREST

The authors report no conflict of interest.

## ETHICAL APPROVAL

This study is based on anonymous data that can be downloaded from a publicly available source. Therefore, no ethical approval is required.
